# Effects of a Nutritional Supplement on Cognitive Function in Aged Dogs and on Synaptic Function of Primary Cultured Neurons

**DOI:** 10.3390/ani9070393

**Published:** 2019-06-27

**Authors:** Maria Elena Pero, Laura Cortese, Vincenzo Mastellone, Raffaella Tudisco, Nadia Musco, Anna Scandurra, Biagio D’Aniello, Giuseppe Vassalotti, Francesca Bartolini, Pietro Lombardi

**Affiliations:** 1 Department of Veterinary Medicine and Animal Productions, University of Naples Federico II, Via Delpino 1, 80137 Naples, Italy; 2 Department of Pathology, Anatomy and Cell Biology, Columbia University, New York, NY 10032, USA; 3 Department of Biology, University of Naples Federico II, Via Cinthia 21, 80126 Naples, Italy

**Keywords:** dog, aging, cognitive disorders, nutraceuticals, spatial navigation test, cFOS

## Abstract

**Simple Summary:**

We tested the effects of a nutraceutical product, DiSenior^TM^, by spatial navigation test and by in vitro and in vivo experiments. Results showed that DiSenior^TM^ was safe and able to ameliorate cognitive functions in aged dogs, as demonstrated by the better performances in the treated with respect the untreated groups. The increase of cFOS, a functional marker of activity in cultured neurons, indicated a positive effect of the substance on neuronal functions. The study suggests that DiSenior^TM^ can improve the quality of life of elderly dogs and may slow the onset of cognitive dysfunction symptoms associated with aging.

**Abstract:**

The objective of this research was to investigate the efficacy of DìSenior^TM^, a nutraceutical formulated to improve cognitive functions in elderly dogs. To this purpose, some clinical and metabolic investigations and a spatial navigation test were performed in treated and untreated dogs. Moreover, the nutraceutical was also tested on primary hippocampal neuron cultures. Results showed no adverse effects on the dogs’ health and a positive effect on learning. In vitro effects on neuron cultures showed an increase in the level of cFOS in treated neurons compared with the vehicle, suggesting that DiSenior^TM^ has also a positive effect on neuronal functions. Overall, this study suggests that DiSenior^TM^ can exert a beneficial effect on aged dogs by preventing the negative effects of aging on cognition. Further studies are needed to assess the mechanisms by which it acts on neurons and the specific effect of the different components alone or combined.

## 1. Introduction

The life expectancy of dogs has significantly increased in recent years, mainly thanks to the progress made by veterinary medicine as well as to improvements in nutrition, elimination of many infectious diseases, better hygiene and, above all, a higher attention to the needs of the elderly patients. For these reasons, an increasing prevalence of a canine geriatric population has occurred, and, in addition to predictable pathologies linked to the aging (heart and kidney failure, osteoarthrosis, neoplastic diseases), a significant increase in so-called “behavioural disorders” has been registered. Canine cognitive dysfunction (CCD) is a progressive, neurodegenerative disease of aged dogs that causes behavioural changes, impaired learning and memory, awareness and confusion [1]. In some studies, CCD has been shown to share certain clinical and neuropathologic characteristics with early human Alzheimer’s disease (AD), for example, diffuse beta-amyloid plaques [2,3]. The decline of cognitive functions in aged dogs was demonstrated in various contexts. Compared to middle-aged dogs, the aged dogs showed a worsening of performance in learning, memory, reversal learning in different cognitive tests [4,5,6,7]. 

The most prevailing signs in dogs affected by CCD have been described by the acronym DISHAA (Disorientation, Interactions with people or other pets, Sleep-wake cycles, House soiling and loss of other learned behaviours, Activity levels (increased or decreased), and Anxiety [1,8,9,10,11,12,13]. Changes may also be seen with self-hygiene (i.e., increased or reduced), appetite or drinking behaviours, and response to stimuli (i.e., exaggerated or reduced) [14]. 

CCD is diagnosed by exclusion of any medical and primary behavioural conditions (neurological disease, sensory decline, musculoskeletal and vascular disease, endocrine, metabolic and degenerative disorders), whose symptoms mimic CCD and which might be a cause of the signs [1,8,12,15,16]. In addition, the presence of several concurrent medical issues may confound a CCD diagnosis. Once signs of CCD are identified, the diagnostic workup should include a thorough medical history, the use of a cognitive screening questionnaire, complete physical and neurologic examination, and laboratory tests (complete blood count, serum biochemistry profile, thyroid hormone levels, and urinalysis). Further diagnostic tests including endocrine testing, radiographs, ultrasound, and magnetic resonance imaging (MRI) could be useful. MRI might show signs compatible with a diagnosis of CCD, but it is primarily indicated for ruling out other intracranial pathology that may mimic CCD [17,18,19,20]. In any event, the definitive diagnosis of CCD can only be obtained via a post-mortem neuropathological examination of the brain.

CCD cannot be cured, but palliative options exist that can reduce disease progression [21]. The treatment options available are diet, supplements and drugs. Moreover, some studies demonstrated that enrichment in the form of mental stimulation and physical exercise can improve quality of life of affected dogs and delay the onset or slow the decline of both the behavioural signs and pathologies associated with brain aging, particularly if combined with an appropriate nutritional base [22,23,24,25,26,27]. Regarding the pharmacological treatment, selegiline hydrochloride is the only pharmaceutical that is approved for the treatment of CCD in aged dogs. Selegiline is as a selective irreversible inhibitor of monoamine oxidase B, although its mechanism of action in dogs is not entirely clear. Selegiline has demonstrated an improvement of the clinical signs of CCD and an improvement in working memory in a laboratory model at a dose of 0.5–1 mg/kg in the morning [28,29,30]. Some dogs improve within the first 2 weeks, while a few do not show improvement until the second month [30]. Other options including propentofylline, a xanthine derivative which may improve microcirculation and inhibit platelet aggregation and thrombus formation to increase oxygenation to the brain and periphery and nicergoline, an alpha 1 and alpha 2 agonist, which may act to enhance cerebral circulation and enhance neuronal transmission and may have a neuroprotective effect [31,32]. 

In addition to pharmacological intervention, natural supplements may also be used and, in recent years, their use in veterinary medicine showed a sharp increase. Generally, supplements have potentially fewer side effects and their use is not contraindicated with most drugs or disease processes (e.g., renal, hepatic, or cardiac dysfunction) in older dogs [33]. 

DìSenior^TM^ is a commercial nutraceutic that includes several substances (e.g., Krill oil and powder, *Boswellia serrata* L.*, Harpagophytum procumbens* L. and *Ginkgo biloba* L.) that have been reported as acting on neuronal integrity and transmission, whereby improving cognition and or preventing cognitive decline in elderly dogs. Krill oil is rich in polyunsaturated fatty acids (n-3 PUFAs), which has various biological activities. Clinical data and animal experiments have established that n-3 PUFAs are involved in maintaining a healthy brain, enhancing brain functions such as memory and learning [34], reactivity, attention and cognitive performance [35]. *B. serrata* L. is a species of trees known for their fragrant resin that has many pharmacological uses, particularly acting as an anti-inflammatory in the cerebrovascular system. Indeed, it significantly reduces neurological deficit, brain infarction, neuronal cell loss and apoptosis in rats [36]. Products of the maidenhair tree, *G. biloba* L., have long been used in China as a traditional medicine for various disorders of health. Its extracts are widely used in the West for the treatment of a wide range of dysfunctions in humans, including memory and concentration problems, confusion, depression and anxiety [37].

The objective of this research was to investigate the effects of DìSenior^TM^ in elderly dogs. Particularly, we aimed to asses: 1. potential adverse metabolic effects; 2. effectiveness on learning and reversal learning skills; and 3. in vitro effects on primary hippocampal neuron cultures. In view of the beneficial activities of the above-mentioned substances, an improvement of cognitive functions in dogs was expected.

## 2. Materials and Methods 

Dogs were enrolled with the owners’ consent. The study was performed on household dogs to avoid any possible interference dependent on usual environment change. DìSenior^TM^ (Dynamopet, Verona, Italy) is a mixture preparation of Krill Oil (100%) 300 mg, Glucosamine sulphate 250 mg, a common polypore mushroom (*Trametes versicolor*) 150 mg, Krill powder 100 mg, *B. serrata Roxb. ex Colebr.* 50 mg, *Harpagophytum procumbens DC* dry extract root 40 mg, *G. biloba* L. leaves dry extract 40 mg, Q10 Coenzyme 30 mg, Vitamin E (RRR-alfa-tocoferil acetate) 24 mg.

Spatial navigation paradigms can be considered valid tools for the practical evaluation of cognitive functions in dogs [38,39,40,41]. They are useful for studying learning, memory and reversal learning in spatial navigation tasks and have been shown to be suitable for studying the effects of senescence in dogs [7,42]. The experiment, including owners’ informed consent, housing, treatment and sampling, was approved by Ethical Animal Care and Use Committee of the University of Naples Federico II, (OPBA, CSV, University of Naples Federico II, prot. PG/2018/00024886) in accordance with local and national law, regulations and guidelines. This study avoided discomfort to the animals using proper clinical management.

### 2.1. Animals

Twenty-two aged dogs of different breeds were involved in this experimental study. Dogs aged between 10 and 17 years (mean age ± SD: 13.4 ± 1.97 years), of different breeds, six females (five spayed) and 14 males (four neutered), were recruited in this experimental study from the client-owned referral population of the Veterinary Teaching Hospital, Department of Veterinary Medicine and Animal Productions (University of Naples Federico II). In each enrolled dog, a clinical and neurological examination and a haematology test including complete blood count, serum biochemistry and thyroid profiles (Thyroid Stimulating Hormone-TSH, total Thyroxine-T4, and free T4) was performed. The health status of each dog was assessed, excluding neurological diseases and endocrinopathies such as hypothyroidism and Cushing syndrome, capable of influencing the dog’s behavior. Questionnaires developed [14] regarding canine behavioral changes were given to the owners. The dogs were randomly assigned in two groups, according to the type of treatment they had to undergo (i.e., supplement or placebo). It was projected to balance the number of samples in the groups. However, some owners did not attain treatment or renounced. The final sample included eleven dogs (13.36 ± 2.14 years; two spayed and one intact females, two neutered and six intact males) in the supplemented group (SG) and nine dogs (13.72 ± 1.58 years; three spayed females, two neutered and four intact males) in the placebo group (PG). No difference was found between the two groups for the mean age (t = 0.80; *p* = 0.44). Following the manufacturer’s suggestion, the treatment (i.e., supplement or placebo) had a total duration of 50 days, divided into two periods of 20 days, separated by 10 days of break. Dogs were subjected to the spatial navigation test, before (round 1) and after (round 2) the treatment period. All tests were conducted at the Laboratory of Canine Ethology (Department of Biology, University of Naples Federico II) in a room of about 4 × 3 m. 

### 2.2. Clinical Scores, Haematology and Serum Biochemistry

The questionnaire focused mainly on: appetite, drinking, active incontinence, day/night rhythm, aimless behavior, activity/interaction, loss of perception, disorientation, memory, personality changes. The total score of all enrolled dogs was > 10 (the lowest total score which can be obtained in this questionnaire is 10 and equals a normal cognitive stage). The clinical signs scores were statistically evaluated using the Wilcoxon non-parametric test PROC NPAR1WAY (SAS, North Carolina, USA). Blood samples were collected before and after the trial from the jugular vein into tubes with and without ethylenediaminetetraacetic acid (K3EDTA), and immediately transported to the laboratory at the Department of Veterinary Medicine and Animal Production, University of Naples Federico II. A Complete blood count (CBC) was performed using a semi-automatic cell counter (Genius S, SEAC Radom Group, Calenzano, Italy). In addition, May–Grünwald–Giemsa-stained blood smears were evaluated for additional information and eventual evidence of platelet clumping. Serum was obtained by centrifugation at 1200× g for 15 minutes, divided in aliquots and frozen at −80 °C. Blood chemistry analyses on serum aliquots were performed by an automatic biochemical analyser (Autolab, AMS Corporation, Rome, Italy) using reagents from Spinreact (Girona, Spain) to determine: haematocrit (HCT), haemoglobin (HB), red blood cells (RBC), white blood cells (WBC), platelets (PLT), total proteins (TP), urea (UREA), creatinine (CREA), glucose (GLU), alanine-aminotransferase (ALT), total bilirubin (BIL), alkaline phosphatase (ALP), cholesterol (CHOL) and triglycerides (TRI). An AIA-360 Automated Immunoassay Analyzer and reagents from Tosoh (San Francisco, CA, USA) were used to assay TT_4_ and fT_4_, TSH was assayed by the Immulite^®^ 2000 Canine (Siemens Medical Solution Diagnostics, Los Angeles, CA, USA). 

### 2.3. Spatial Navigation Test

All dogs were subjected to the spatial navigation test to determine their learning and reversal learning skills.

#### 2.3.1. Determination of Navigation Strategy

To determine the preferred strategy in a spatial navigation task the experimental protocol included two phases: the learning phase, where dogs had to learn the position of a food bowl within a plus maze, and the strategy phase, where dogs could reach food both by adopting an egocentric or allocentric strategy. To determine the preferred strategy of dogs to solve the task, the two phases described above were administered one after the other two or three times (see details below).

##### 2.3.1.1. Experimental Setting

The plus-shaped maze was realized with plastic panels 2.0 m high and placed inside the experimental room. All arms of the maze were 0.8 m long, 0.60 m wide and had a door at their end. The central area of intersection between the four arms had a dimension of 0.60 × 0.60 m. The maze flooring was covered with grey polyvinyl chloride flooring (PVC). The spatial references provided to the dogs to orient themselves had been placed inside the maze: alternating strips of grey and black PVC were arranged in triangle shape on the floor across one of the two axes of the maze and a 0.50 m high stake was placed at the 0.50 m from the base of the triangle. Two empty bowls were placed in the two opposite arms lateral to the starting point. All the elements outside the maze (e.g., room door, walls, etc.) were covered by white curtains to prevent dogs from using them as spatial references.

##### 2.3.1.2. Familiarization

The aim of this procedure was to familiarize the dogs with all the elements inside the experimental room. Dogs and owners entered the experimental room accompanied by the experimenter. For about 5 minutes the dogs were left free to explore the space outside the maze. Subsequently, the two doors of the maze positioned at the base and apex of the PVC triangle were opened by the experimenter. The experimenter asked the owners to enter the maze with their dogs, starting from a door and exiting the opposite one, then returning and repeating the reverse path. As soon as the owners and the dogs came out of the maze, the experimenter closed the maze.

##### 2.3.1.3. Side Choice Trial

The aim of this trial was to standardize the position of the food inside the maze (i.e., in the east or west side of the entrance door) depending on the dogs’ first choice. After opening the entrance door of the maze, the experimenter asked to owners to release the dogs inside the maze. In this trial there was no food inside both bowls. As soon as the dogs chose the empty food bowl in one of the two lateral arms, the owners reached the dogs inviting them to exit the maze before they dogs could explore the opposite lateral arm. The arm not chosen by the dogs was then classified as the correct arm (where the baited food bowl was placed) for the next learning phase.

##### 2.3.1.4. Learning Phase

**Figure 1:**
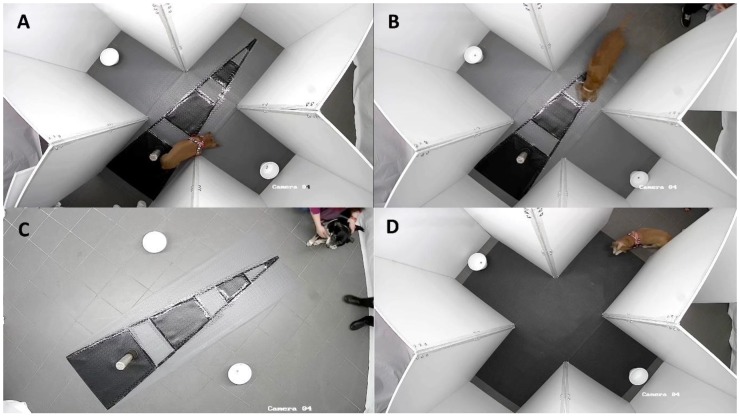
The figure shows the different phases of the spatial navigation test. (**A**) Learning phase, (**B**) Strategy phase, (**C**) Reversal learning phase (Allocentric), (**D**) Reversal learning phase (Egocentric).

The goal of this phase was that the dogs learn the position of the correct bowl (i.e., the bowl with reachable food), choosing it repeatedly in a consecutive series of trials. Before starting each trial, the experimenter put one piece of food (i.e., sausage) inside one of the two bowls. The empty bowl was placed in the opposite arm of the maze (Figure 1A). To balance the odour and prevent the dogs from choosing based on the odour cues, a piece of food was positioned below the incorrect bowl, in a space not reachable by the dogs. Each trial started when the experimenter opened the entrance door of the maze and the owners and dogs were in position standing at the base of the PVC triangle. The experimenter gave a verbal signal (i.e., the word “ok”) to the owners to release the dogs. When the dogs reached one of the two bowls in the side arm, the owners went to the central part of the maze to bring the dogs out, thus preventing the dogs from approaching the bowl in the unselected arm. The trial was classified as correct if the dogs entered the correct arm; otherwise, the trial was classified as incorrect. The criterion to pass this phase was of three consecutives correct trials.

##### 2.3.1.5. Strategy Phase

The objective of this phase was to identify the dog’s navigation strategy to reach the correct bowl when they entered the maze from the opposite side respect to that of the learning phase: dogs could adopt an allocentric strategy using the spatial relationship between the cues inside the maze to orient themselves, or they could adopt an egocentric strategy choosing to repeat the same motor response (i.e., turning on the left or the right from the starting point) used in the previous learning phase. The experimental procedure was the same as the learning phase but consisted of only one trial and the food was placed in both bowls in the two lateral arms (Figure 1B). The entrance door in the strategy phase was the opposite of the previous phase. 

##### 2.3.1.6. Classification of the Preferred Strategy

The classification of the preferred strategy of the dogs was based on the results obtained after the consecutive repetitions of the learning and the strategy phases: if in the second repetition the dogs chosen the same strategy as the first repetition, they could be classified as “allocentric” or “egocentric” according to the type of strategy adopted. If the strategies adopted in the two repetitions were different, we proceeded with a third repetition of the learning and strategy phases. In the latter case, the classification was based on the strategy adopted for two repetitions out of three. After the classification of the strategy, the dogs passed to the next phase after an interval of about 15 minutes in which the owners were asked to take a walk with the dogs.

#### 2.3.2. Reversal Learning Phase 

The purpose of this phase was to evaluate the cognitive flexibility of dogs by forcing them to use the non-preferred navigation strategy (i.e., opposite to that adopted in the previous learning phase) to reach the food. For this purpose, the experimental setting was arranged so as not to allow the resolution of the task using the preferred strategy of the dogs (see the details in the following paragraphs). During this phase, the dogs were subjected to a minimum of 16 trials to reach the success criterion of three correct consecutive choices, with a maximum of 32 trials. If the dogs did not reach criterion in the 32 trials, the test ended. The position of the food for each dog in this phase was established using the same procedure of the side choice trial (see Section 2.3.1.3), adapted to the experimental setting of the reversal learning phase.

##### 2.3.2.1. From Egocentric to Allocentric Strategy

To force the dogs classified as egocentric to use the allocentric strategy (i.e., using the cues to solve the task), the experimental setting was reorganized: the walls of the maze were eliminated and only the PVC floor with the triangle and the stake were left in the room. The two bowls were placed in a medial position to the east and west of the PVC triangle base (Figure 1C). At each trial, the starting point was alternated between the base and the apex of the PVC triangle, so that the dogs would refer to the relative position of the cues space to choose the correct bowl, rather than turning to the right or left of the starting point. When the owners and the dogs were stationary at the starting point, the experimenter gave a verbal signal (i.e., the word “okay”) to the owners for the release of the dogs. As soon as the dogs chose one of the two bowls, the owners quickly carried the dogs out of the experimental room, thus preventing the dogs from approaching the unchosen bowl. If the dogs chose the bowl with food, the trial was classified as correct; alternatively, it was considered incorrect. To prevent a possible orientation from the entrance door of the room, all the elements (i.e., PVC floor, stake and bowls) were rotated inside the room in each trial, maintaining the same relative positions.

##### 2.3.2.2. From Allocentric to Egocentric Strategy

To force the dogs classified as allocentric to use the egocentric strategy (i.e., turning on the left or the right from the starting point) all the cues used in the learning phase (i.e., PVC triangle on the floor and the stake) were removed. The food was placed always in the same relative position (i.e., on the east or west side) from the entrance door (Figure 1D). In this phase all doors of the maze were used, alternating the four different doors of the maze as the starting point in each trial. 

#### 2.3.3. Data Collection and Analysis

The spatial navigation tests were video recorded and analysed with the Solomon Coder beta^®^ 14.05.19 (ELTE TTK, Budapest, Hungary). All data about to the type of choices (i.e., correct or incorrect) in the learning and reversal learning phases, the number of trials to reach the criterion and the type of strategy used in the strategy phase were collected for the analyses. A second observer collected the same data for the inter-observer reliability and there was no difference between the two observers. To verify the normal distribution of the data, the D’Agostino and Pearson omnibus normality test was performed. A non-parametric statistic was adopted for data that did not pass the normality test. Comparisons were made between the two experimental groups (i.e., supplement vs. placebo) and, in each group, between the data obtained before and after treatment (i.e., round 1 vs. round 2). All analyses were performed with GraphPad Prism^®^ software 5.01 (GraphPad Software Inc., San Diego, CA, USA).

### 2.4. Primary Hippocampal Neuron Cultures

Primary embryonic hippocampal neurons were cultured as previously described [43,44]. Briefly, hippocampi were dissected from E18 rat embryos, and neurons plated on 100 μg/mL poly-D-lysine-coated 12-well-plates at the density of 3 × 10^5^ cells/well for biochemistry assays, 5 × 10^4^ cells/dish, or 4 × 10^4^ cells/coverslip on 18 mm coverslips for DiOlistic Labeling. Primary neurons were maintained in Neurobasal medium (Invitrogen) with the supplement of 2% B-27 (Invitrogen, Thermo Fisher Scientific, Waltham, MA, USA) and 0.5 mM glutamine (Invitrogen, Thermo Fisher Scientific, Waltham, MA, USA) and 1/3 of medium was changed every 3–4 days up to 4 weeks in culture.

#### 2.4.1. β-Amyloid_42_ and DìSenior^TM^ Preparation

Oligomer-enriched preparations of Aβ were obtained according to previously published methods [44]. Lyophilized Aβ (rPeptide, Whatkinsville, Georgia, USA) was equilibrated to room temperature for 30 min to avoid condensation upon opening the vial. The lyophilized peptide was resuspended in 1,1,1,3,3,3-hexafluoro-2-propanol to a concentration of 1 mM for another 2 h at room temperature and then aliquoted into low protein–binding Eppendorf tubes. Monomeric Aβ aliquots were dried under vacuum in a SpeedVac and stored at −80 °C. To prepare oligomer-enriched preparations, the aliquots were resuspended in anhydrous dimethyl sulphoxide (DMSO) (Sigma-Aldrich, Saint Louis, MI, USA) to 5 mM followed by vortexing and 10 min sonication. The resuspended peptide was diluted to 100μM in ice-cold Ham’s F-12 medium, immediately vortexed for 30 s, and then incubated at 4 °C for 24 h before use. Total Aβ concentration was measured by bicinchoninic acid assay (BCA) after oligomerization, and 10 μM of oligomeric Aβ was used in all experiments unless otherwise noted. Scrambled Aβ control (rPeptide) was prepared using same protocol mentioned earlier in this section. To form Aβ_42_ aggregates stocks of 1 mM peptide were resuspended in PBS to a concentration of 100 μM and incubated at 37 °C for 24 h. A concentration of 10 μM Aβ_42_ was used in all experiments unless noted otherwise. DìSenior^TM^ preparation was diluted in 500 mL of Neurobasal media and filtered with 0.22 μm filter. 100 μL were added for 24 h to the neurons. Filtration was a necessary step because the mixture preparation is enriched of non-soluble components.

#### 2.4.2. DiOlistic Labeling 

Hippocampal neurons were fixed with 4% PFA for 10 minutes at room temperature and washed three times with 1Å ~ PBS subsequently subjected to DiOlistic labeling. The Helios gene gun system (Bio-Rad, Haercules, CA, USA) was used according to the manufacturer’s instructions. Tungsten particles (1.1 μm, Bio-Rad, Haercules, CA, USA) coated with DiI (Invitrogen, Thermo Fisher Scientific, Waltham, MA, USA), which defines the neuronal architecture in red, were delivered into hippocampi sections. Coverslips were mounted in ProLong Gold antifade reagent (Invitrogen, Thermo Fisher Scientific, Waltham, MA, USA) and imaged the next day using an Olympus IX83 microscope (Olympus Corporation, Shinjuku, Japan) with the wide field and disk-scanning unit spinning-disk. 

For the immunoblotting of cFOS (a proto-oncogene expresses in neuron and a marker of synaptic plasticity), whole cell lysates from control and treated cultured hippocampal neurons were obtained by manual homogenization in Ripa buffer containing protease inhibitors (Thermo Fisher Scientific, Waltham, MA, USA) followed by sonication (Bransor-Sonifier 250 for 30 sec). Protein concentration was determined using the Bio-Rad Laboratories protein assay kit (Bio-Rad, Haercules, CA, USA). Samples were boiled (95 °C for 5 min) and pre-cleared (5 min at 10,000× g) prior to loading and transfer. Equal amounts of protein were separated on 12% polyacrylamide gels. Protein bound nitrocellulose membranes were probed for cFOS (rabbit, 1:500), Millipore GADPH (mouse and rabbit, 1:5000). Secondary antibodies were conjugated to IR680 or IR800 (Rockland Immunochemicals, Pottstown, Pennsylvania, USA) for multiple infrared detection. Image acquisition was performed with an Odyssey imaging system (LI-COR Biosciences, Lincoln, NE, USA).

## 3. Results

### 3.1. Clinical Scores and Biochemical Analysis

Clinical scores where superimposable at the time 0 (SG = 10.13 *vs* PG = 10.94, respectively), but a difference was detected at the end of the supplement administration (SG = 8.81 *vs* PG = 12.55, respectively). However, no statistical difference between treated and untreated dogs either before nor after the trial was detected. All haematological and biochemical parameters fall in the normal range for adult dogs and no statistical differences emerged between groups and no time effect was recorded (Table 1).

### 3.2. Spatial Navigation Test

#### 3.2.1. Determination of Navigation Strategy

Dogs in the SG reached the criterion in the first learning task in an average (± SD) of 9.55 ± 8.50 trials for the round 1 and 5.64 ± 2.77 trials for the round 2. In the second repetition of the learning task, dogs used 7.27 ± 4.41 for the round 1 and 5.66 ± 3.41 trials for the round 2. Two dogs needed a third repetition of the learning to classify their strategy in the round 1, reaching the criterion in three trials; otherwise, four dogs needed third learning repetition in the round 2 with an average of 4.75 ± 2.06 trials. For the PG, dogs reached the criterion in the first learning task in an average of 14.89 ± 14.70 trials for the round 1 and 13.78 ± 9.01 trials in the round 2. The average of 4.33 ± 1.80 trials in round 1 and 6.78 ± 5.17 in round 2 were needed in the second repetition of the learning. Four dogs of the PG in round 1 needed a third learning repetition to classify their strategy with an average of 6.75 ± 4.34 trials, whilst two were classified after the third learning repetition with an average of 7.50 ± 4.95 trials. There is no difference between groups in the round 1 for each repetition of the learning phase. In the round 2, the two groups differed in the number of trials to reach the criterion in the first learning task with a lower number in the SG compared to the PG (Mann-Whitney test, U = 14.5, *p* < 0.01). 

**Figure 2:**
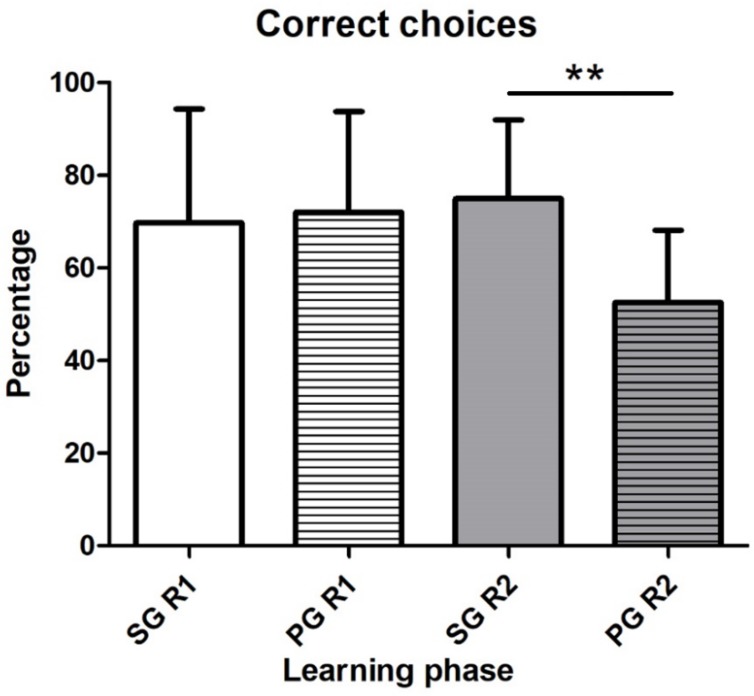
Mean ± SD of the percentage of correct answers in the Learning phase. R1: Round 1, R2: Round 2. SG: supplement group, PG: placebo group. *p* value: ** *p* < 0.001.

Considering overall the learning phase (i.e., including all the repetitions of the task), the dogs of the SG passed the phase with percentages of correct choices of 69.76 ± 24.57 % in round 1 and 74.99 ± 16.96 in the round 2. For the PG, the percentages of correct choices were 71.97 ± 21.81% in the round 1 and 52.49 ± 15.68% in the round 2. There was no difference between two groups in the round 1, whereas in round 2 the SG made more correct choices in percentage than PG (Mann–Whitney U = 13.00, *p* < 0.01; Figure 2). Compared to the performances in round 1, the PG showed a tendency to decrease correct choices in percentage for the learning phase in round 2 (Wilcoxon test, W = 33.00, *p* = 0.05).

#### 3.2.2. Reversal Learning Strategy

All dogs completed successfully the reversal learning phase in round 1 and 2, except for one dog in the SG and one dog in the PG that did not reach the criterion respectively in round 1 and 2. In the SG, dogs reached the criterion in an average of 9.50 ± 8.45 trials in round 1 and 10.09 ± 6.35 trials in round 2. Dogs of the PG needed 9.11 ± 6.59 trials in round 1 and 11.13 ± 4.39 trials in round 2 to reach the criterion. No difference was found between the two groups both before and after treatment for the number of trials to reach the criterion. Globally, the percentage of correct choices in round 1 was 59.66 ± 25.40 for the SG and 61.81 ± 12.28 for the PG. In round 2, the percentage was 63.64 ± 17.41 for SG and 52.31 ± 20.56 for the PG. There was no significant difference between groups in both round 1 and 2. 

### 3.3. Effects on Cultured Hippocampal Neurons

**Figure 3:**
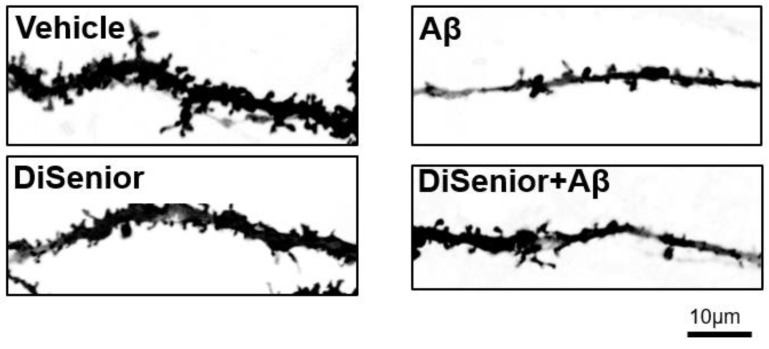
Disenior^TM^ does not ameliorate loss of spine density induced by Amyloid-Beta.

Hippocampal neurons treated with 100 uLof 5000× diluted and filtered DìSenior^TM^ preparation (10gr) for 24 h, showed that DìSenior^TM^ did not induce any change in dendritic spines density (Figure 3, vehicle vs. DìSenior^TM^). Both vehicle and treated neurons appeared morphologically similar. Moreover, hippocampal neurons incubated with Aβ 10uM for 24 h in the presence of DìSenior^TM^ preparation showed that it did not induce a significant ameliorative effect of the loss of spines induced by Aβ (Figure 3). 

**Figure 4:**
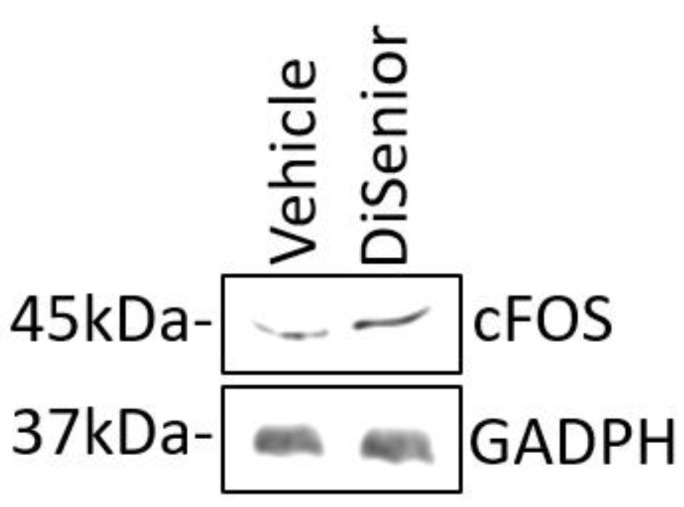
Disenior^TM^ increases neuronal activity. Immunoblot of cFOS level in hippocampal neurons (21DIV) treated with Disenior^TM^.

Finally, an increase in the level of cFOS was observed in treated neurons compared with vehicle (Figure 4).

## 4. Discussion

In this research we tested for the effect of a nutraceutical product in vitro and in vivo experiments. In both cases some ameliorative effect was observed. All haematological and biochemical parameters fall in the normal range for aged dogs, which confirms that no occasional diseases occurred during the interexperimental phases in any dog and, more importantly, the nutritional supplement was well tolerated and did not cause any adverse effect.

With regard to the spatial navigation test, the results obtained in the learning phase showed a positive effect with dogs of SG, showing a lower number of trials to reach the criterion and a higher percentage of correct choices compared to PG at the end of the treatment (round 2). These results indicate that the treatment prevents the general decrease in learning due to the aging process [4,27]. On the other side, no significant difference between groups in both round 1 and 2 in reversal learning was recorded. Reversal learning measures the cognitive flexibility, which tend to worsen in aged dogs [7]. It has been proposed that the deterioration of cognitive functions could be a result of an increased behavioral rigidity [4]. The prefrontal cortex and the hippocampus, two areas of the brain implicated in spatial memory and cognitive flexibility, are among the areas most affected by neurodegenerative processes related to aging [45]. It is possible that reversal learning could not benefit from the treatment because it is a complex process, maybe requiring longer treatment times. In effect, an ameliorative trend in SG was observed.

Our results showed that DìSenior^TM^ did not induced significant changes in hippocampal neurons, either in dendritic spines density, or on its morphological structure likely for the same reason of the short time treatment (24 h). Nevertheless, because in dogs we observed at the end of the treatment, a trend of improvement in behavioral task performance, neuronal activity on primary cultured hippocampal neurons 21 days in vitro (DIV) was investigated by checking the level of cFOS. Recent studies demonstrated that c-FOS-expressing neurons are involved in the formation of memory engrams [46,47,48]. Here, we found an increase in the level of cFOS in treated neurons compared with the vehicle, suggesting that DiSenior^TM^ has a positive effect on neuronal functions.

Taken together, the results suggest that DìSenior^TM^ had no aversive effect on learning or physiology. Rather, it showed beneficial effects in preventing the cognitive decline of aging in dogs. Further studies are needed to assess the mechanisms by which it acts on neurons.

## Figures and Tables

**Table 1:** Haematological, biochemical and hormonal traits in supplemented and placebo groups before and after the treatment period (rounds 1 and 2, respectively). Results were subjected to the analysis of variance JMP (9.0) of SAS (2000). Tukey's test was adopted as a multiple-comparison test to determine the source of variation. HCT—haematocrit; HB—haemoglobin; RBC—red blood cells; WBC—white blood cells; PLT—platelets; TP—total proteins; Crea—creatinine; GLU—glucose; ALT—alanine-aminotransferase; BIL—total bilirubin; CHOL—cholesterol; TRI—triglycerides; TSH-thyroid stimulating hormone.

Item	Unit	GROUP				P		RMSE
		Supplement (SG)		Placebo (PG)				
		Round 1	Round 2	Round 1	Round 2	GROUP	TIME	
HCT	%	40.5	40.4	41.8	41.1	0.282	0.713	2.98
HB	g/dL	15.2	14.9	15.3	15.8	0.435	0.938	2.03
RBC	10^6^/mL	7.0	6.7	7.1	6.8	0.652	0.314	0.992
WBC	10^3^/mL	9.1	9.4	8.3	8.6	0.099	0.504	1.47
PLT	10^3^/L	275.4	285.8	287.4	273.6	0.994	0.971	44.4
TP	g/L	6.4	6.4	6.5	6.5	0.766	0.081	1.1
Urea	mg/dL	41.5	39.7	47.7	50.3	0.087	0.959	14.76
Crea	mg/dL	1.4	1.3	1.2	1.2	0.780	0.111	0.009
GLU	mg/dL	94.2	93.4	92.3	93.1	0.888	0.868	12.31
ALT	U/L	55.1	72	54.7	53.0	0.384	0.440	34.69
ALP	U/L	223.3	224.1	242	249	0.652	0.944	155
BIL	mg/dL	0.3	0.3	0.1	0.1	0.059	0.808	0.296
CHOL	mg/dL	188.1	182.5	185.3	209.4	0.136	0.796	49.88
TRI	mg/dL	59.9	58.2	72.4	71.2	0.087	0.837	22.92
TT_4_	g/dL	2.1	2.1	2.3	2.2	0.360	0.888	0.446
fT_4_	ng/dL	1.1	1.2	1.3	1.3	0.151	0.578	0.351
TSH	IU/L	22.7	23.4	24.7	21.8	0.918	0.579	5.37

RMSE: root mean square error.
